# Advancing Reliable Data for Cancer Control in the Central America Four Region

**DOI:** 10.1200/JGO.2016.008227

**Published:** 2017-03-08

**Authors:** Marion Piñeros, Silvina Frech, Lindsay Frazier, Mathieu Laversanne, Joaquin Barnoya, Claudia Garrido, Eduardo Gharzouzi, Andrea Chacón, Soad Fuentes Alabi, Lisseth Ruiz de Campos, Jacqueline Figueroa, Ricardo Dominguez, Ofelia Rojas, Rosario Pereira, Carla Rivera, Douglas R. Morgan

**Affiliations:** **Marion Piñeros** and **Mathieu Laversanne**, International Agency for Research on Cancer, Lyon, France; **Silvina Frech**, US National Cancer Institute, Bethesda, MD; **Lindsay Frazier**, Dana-Farber/Children’s Cancer Center, Boston MA; **Joaquin Barnoya** and **Eduardo Gharzouzi**, Instituto de Cancerologia; **Claudia Garrido**, Unidad Nacional Oncología Pediátrica, Guatemala City, Guatemala; **Joaquin Barnoya**, Washington University in St. Louis, St. Louis, MO; **Andrea Chacón**, Unidad Nacional para la Prevención y control del Cáncer, Ministerio de Salud; **Soad Fuentes Alabi**, Hospital Benjamín Bloom, Ministerio de Salud; **Lisseth Ruiz de Campos**, Asociación Salvadoreña para la Prevención del Cáncer, San Salvador, El Salvador; **Jacqueline Figueroa**, Unidad de Registro de Cáncer, Secretaria de Salud, Tegucigalpa; **Ricardo Dominguez**, Hospital de Occidente, Secretaria de Salud, Copán, Honduras; **Ofelia Rojas**, **Rosario Pereira**, and **Carla Rivera**, Universidad Nacional Autónoma de Nicaragua, León, Nicaragua; and **Douglas R. Morgan**, Vanderbilt University, Nashville, TN.

## Abstract

The Central America Four (CA-4) region, comprising Guatemala, Honduras, El Salvador, and Nicaragua, is the largest low- and middle-income country region in the Western Hemisphere, with over 36 million inhabitants. The CA-4 nations share a common geography, history, language, and development indices, and unified with open borders in 2006. The growing CA-4 cancer burden among the noncommunicable diseases is expected to increase 73% by 2030, which argues for a regional approach to cancer control. This has driven efforts to establish population-based cancer registries as a central component of the cancer control plans. The involvement of international and academic partners in an array of initiatives to improve cancer information and control in the CA-4 has accelerated over the past several years. Existing data underscore that the infectious cancers (cervical, stomach, and liver) are a particular burden. All four countries have committed to establishing regional population-based cancer registries and have advanced significantly in pediatric cancer registration. The challenges common to each nation include the lack of national cancer control plans and departments, competing health priorities, lack of trained personnel, and sustainability strategies. General recommendations to address these challenges are outlined. The ongoing regional, international, and academic cooperation has proven helpful and is expected to continue to be a powerful instrument to contribute to the design and implementation of long-term national cancer control plans.

## BACKGROUND AND SIGNIFICANCE

The annual global incidence of cancer is projected to increase from 12.7 to 22.2 million by 2030, with 13.1 million expected deaths. Over two thirds of the burden will occur in low- and middle-income countries (LMICs), wherein seven cancers (lung, colon, breast, stomach, liver, cervical, and esophageal) account for nearly two thirds (62%) of incident cases.^1^

Cancer registration is a crucial element of appropriate planning, implementation, and evaluation of comprehensive cancer control plans. Enormous disparities in the availability, coverage, and quality of the data on cancer burden, and specifically on cancer incidence, exist among countries.^2^ High-quality population-based cancer registries (PBCRs) are the means to obtain cancer incidence, a critical measure for cancer control, but cover only 8%, 6%, and 2% of the populations in Latin America and the Caribbean, Asia, and Africa, respectively.^2^ Civil registration and vital statistics systems are also underdeveloped, and their coverage is < 50% in most LMICs.^3^ Only 34 countries, representing 15% of the world’s population, produce high-quality cause-of-death data (defined as > 90% of deaths are registered and < 10% of deaths are coded with ill-defined signs and symptoms).^3^ Although much of Latin America is lacking high-quality PBCRs, overall, the region has better vital statistics systems in place compared with Africa and Asia.^4^

In Latin America, the cancer burden is projected to grow significantly by 2030, with 1.7 and 1.0 million annual incident cases and deaths, respectively.^1^ This growing cancer burden, with an enormous impact on families and societies, has prompted governments to prioritize cancer control planning. The situation and advancements in cancer control in Latin America and the Caribbean have been reported in two *Lancet Oncology* commissions,^5,6^ and additional brief reports on the advancements in cancer registration are available.^7^ The Central America Four countries (CA-4; Guatemala, Honduras, El Salvador, and Nicaragua) form an important LMIC subregion in the Western Hemisphere, currently understudied regarding cancer epidemiology and control.

International efforts in cancer control in LMICs have markedly expanded since the United Nations General Assembly on noncommunicable diseases (NCDs) in 2011. This was the first General Assembly ever to focus on a non-HIV health issue. Key organizations, such as the International Agency for Research on Cancer (IARC) and the US National Institutes of Health/National Cancer Institute (NCI) responded with the creation of the Global Initiative on Cancer Registry Development (GICR) and the Center for Global Health (NCI-CGH), respectively. The IARC-GICR has prioritized the development of PBCRs in low-resource settings. In the Central America LMICs, the International Cancer Control Partnership, Pan American Health Organization (PAHO), Union for International Cancer Control (UICC), IARC-GICR, NCI-CGH, and academic partners (eg, Vanderbilt University, MD Anderson Cancer Center, Dana-Farber Cancer Institute) have joined together to increase capacity in cancer control and cancer registration.

Nascent efforts in the CA-4 and Central America include (1) the first-ever CA-4 Cancer Control and Bioinformatics Congress (October 2014); (2) IARC site visits in the context of the GICR (http://gicr.iarc.fr/) and the International Atomic Energy Agency (IAEA) imPACT site visits (http://cancer.iaea.org/impact.asp) and recommendations (2014-2016); and (3) the first-ever NCI-CGH Cancer Control Leadership Forum for Central America and the CA-4 (September 2016). Concrete short-term outcomes have included the creation of Ministry of Health (MOH) cancer control divisions and the planning and implementation of adult and pediatric PBCRs in selected CA-4 countries. Herein, we highlight relevant infrastructure features in the CA-4 region and discuss the advancements and key challenges faced to establish PBCRs as a central element of cancer control planning.

## THE CA-4 REGION

The Central America Integration System (SICA), based in El Salvador, was established in 1993 as a cooperative agreement for economic and political goals of the seven republics of Central America.^8^ Thereafter, within SICA, the Council of Ministers of Health of Central America (COMISCA) was formed to serve as the oversight body for regional health policy. COMISCA submits policy and implementation recommendations to the annual Central America Presidential Summit. SICA and COMISCA now include the Dominican Republic. Even before the SICA umbrella, Central America had a history of regional centers of excellence, including the Institute of Nutrition of Central America and Panama (based in Guatemala) and the Panamerican School of Agriculture, Zamorano (based in Honduras). As in much of Latin America, the health delivery sector has three main components: the MOH public facilities, the governmental employee (Seguro Social) facilities, and private institutions. Private institutions include clinics and hospitals and the nongovernmental organization services.

**Fig 1:**
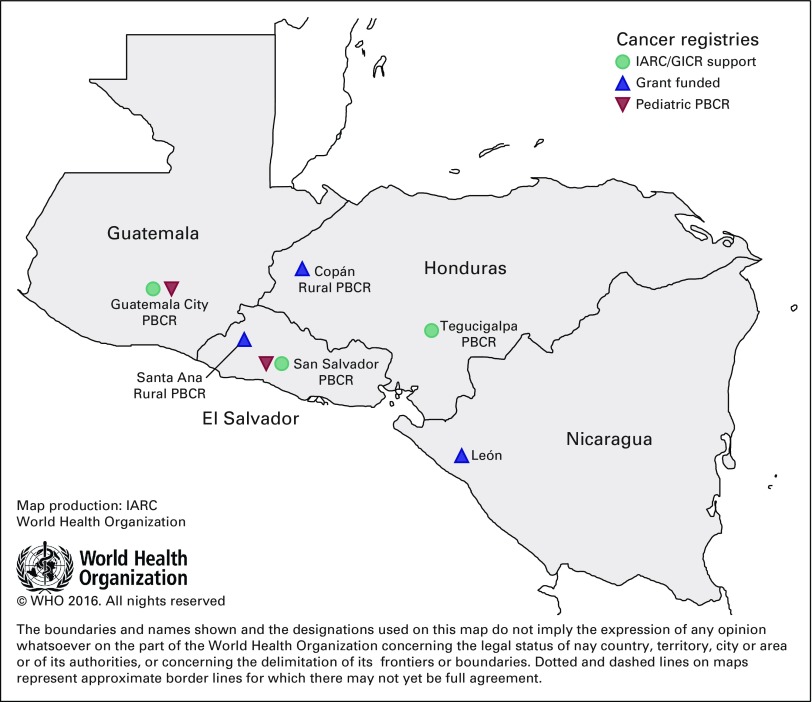
The Central America Four region and nascent cancer registries. Population-based cancer registries (PBCRs) and year of implementation initiation: Guatemala City (2015), Francisco Morazán (Tegucigalpa, 2016), San Salvador (2017), Copán (2014), Santa Ana (2017), and León (2017). Pediatric PBCRs: Guatemala City (2014), and San Salvador (2014). GICR, Global Initiative on Cancer Registry Development; IARC, International Agency for Research on Cancer.

The CA-4 consortium of the four northern countries of Central America are interconnected by geography, history, language, and development indices (Fig 1). In 2006, the CA-4 opened borders, similar to the European Union, and has been in transition toward a union of many aspects of their infrastructure, which has had implications for the health systems, with an appreciable flow of patients across borders. The development indices in the CA-4 rank among the lowest in the Western Hemisphere, and available data suggest that multidimensional poverty approaches 20%.^9^ An important challenge for health programs, cancer registries, and cancer control initiatives is that nearly half (48%) of the CA-4 population lives in rural areas (Table 1). El Salvador is the exception (34%), primarily due to the urban influx during the civil war of the 1980s. The mean health sector and public health expenditures are low, ranging from 6.5% to 8.6% (Table 1), compared with those in Costa Rica (9.9%) and the United States (17.1%).^9^

**Table 1:**
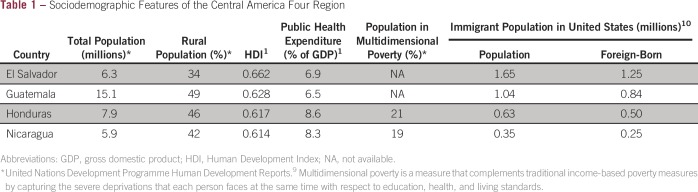
Sociodemographic Features of the Central America Four Region

Noteworthy is that the CA-4 countries account for a large number of the recent immigrant population to the United States, which makes the region unique among global LMICs from a US perspective (Table 1). A number approximately equivalent to one quarter of the population of El Salvador lives in the United States, and most are foreign born. From the US viewpoint, the research and prevention initiatives in the CA-4 may be informative for the US Hispanic population, particularly for those from the region (over 4.5 million).^10^

## THE BURDEN OF CANCER IN THE CA-4 REGION

In the current health profile of the CA-4 region, in certain areas, cancer is a leading cause of morbidity and mortality. Overall, infectious diseases, trauma and injury, nutritional deficiencies, and child and maternal health continue to be the leading challenges for the health systems.^11^ Nevertheless, some CA-4 nations display some of the highest cervical, stomach, and liver cancer incidence rates in the Western Hemisphere, and the number of cancer cases (35,000 annual cases in 2012) in Central America is expected to increase by 73% by 2030.^1^ Importantly, although cancer incidence and mortality rates for US Hispanics are lower overall compared with other racial and ethnic groups, the opposite is true for the infection-associated cancers.^12-15^

**Fig 2:**
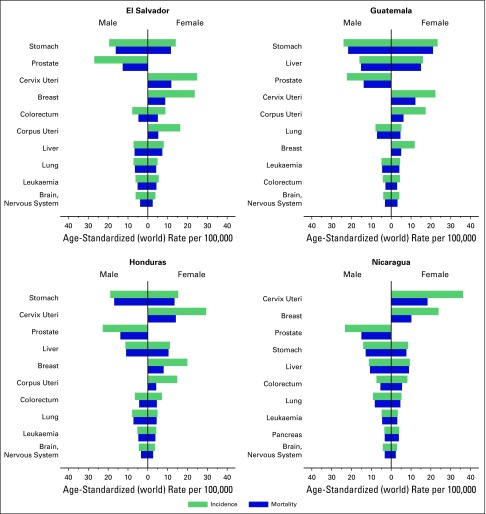
Age-standardized cancer incidence and mortality rates in the Central America Four region

The estimated annual number of incident cancer cases in the CA-4 countries ranges between 5,200 for Nicaragua and 13,000 for Guatemala^1^ (Table 2). The cancers of infectious origin and linked to poverty (stomach, cervical, and liver) are predominant, both in incidence and mortality (Fig 2).^16^ Globally, hepatocellular carcinoma is considered an infectious cancer; however, in the region, additional factors may be important, such as aflatoxin exposure and nonalcoholic fatty liver disease. Western Honduras is a rural region with centralized MOH services, and the Western Honduras Gastric Cancer Initiative has been able to provide population-based estimates of gastric adenocarcinoma incidence, confirming the high rates.^17,18^ A parallel initiative for gastric cancer with similar aims is under way in El Salvador at the national level, also with NCI funding. These initiatives serve as examples of the utility of high-quality single-cancer registry efforts, which also serve as a platform for regional cancer registry capacity building.

**Table 2:**
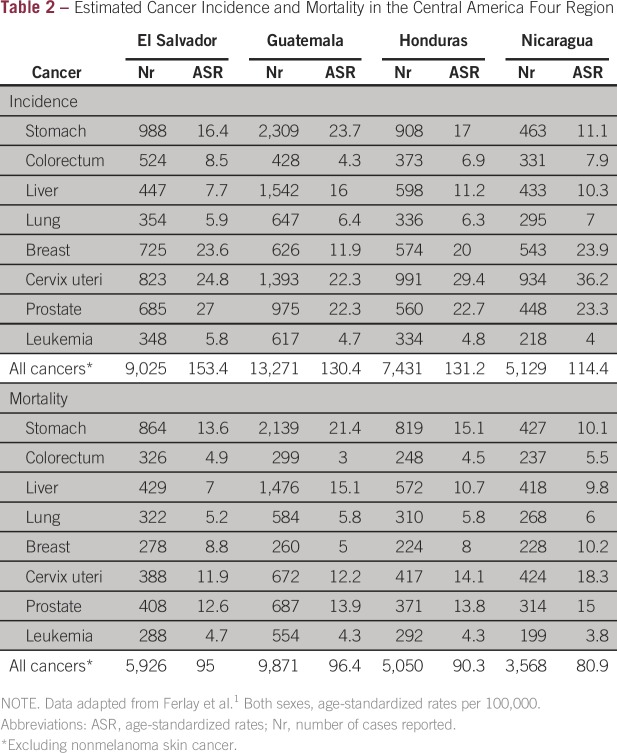
Estimated Cancer Incidence and Mortality in the Central America Four Region

In the absence of PBCRs, caution is advised when using the cancer burden estimates, although they likely are underestimations, given the limitations in the source and quality of the data used.^1^ Also, there may be limited information available on mortality by cause of death, as noted in Honduras, which poses an additional limitation on existing cancer estimates.^19^

Although pediatric cancers represent less than 3% of the estimated new cancer cases, pediatric cancer care in the CA-4 region illustrates the potential for partnerships to significantly affect cancer survival. In less than 20 years, the 3-year event-free survival for standard-risk acute lymphoblastic leukemia in the region has increased from 38% to 68%.^20,21^ One of the chief mechanisms for the dramatic improvement in survival has been the twinning of pediatric cancer facilities in the CA-4 with partners in higher-income countries. In addition, because pediatric cancer is less common and care has been centralized within each country at one or two sites, this twinning model is feasible. In Central America, initiatives began in 1986 with an alliance between La Mascota in Nicaragua and hospitals in Monza and Milan, Italy, and Bellinzona, Switzerland.^22-24^ The partnerships later expanded to include the other Central American countries, as well as other centers in Italy, the United States, and Canada.

In Central America, this joint enterprise eventually led to the creation in 1996 of the Central American Pediatric Hematology-Oncology Association (AHOPCA), a regional association that includes pediatric oncologists and other oncology providers (nurses, surgeons, psychologists, and pathologists). AHOPCA has developed common, resource-adapted protocols for each of the major childhood cancers, implemented across the region, with monitoring of outcomes over time. An important catalyst for AHOPCA has been funding provided by the International Outreach Program of St Jude Children’s Cancer Center that provides salary support (eg, for physicians, nurses, psychologists, and data managers at each site) and support for the annual AHOPCA meeting and related operational initiatives.^25^

The St Jude International Outreach Program also spearheaded the development of the Pediatric Oncology Network Database (POND), a database used to register the patients at each site, monitor delivery of protocol-based care, and document toxicities and survival. POND acted essentially as a hospital-based cancer registry (although International Classification of Diseases of Oncology and International Classification of Childhood Cancer coding was not used) and created a mechanism for the AHOPCA centers to combine their data, monitor quality and outcomes, and then formulate regional recommendations. POND cemented into the AHOPCA culture the value of real-time monitoring of outcomes and quality indicators. The demonstrated utility of the POND has been critical in convincing the CA-4 pediatric oncologists of the value of PBCRs to systematically track incidence and survival and serve as a basis for research into the epidemiology of childhood cancer.

## TOWARD THE DEVELOPMENT OF PBCRS IN THE CA-4

### Advances and Challenges in PBCR Development

In recent years, nascent programs and collaborations between the CA-4 and national and international partners provide insight into the current state and challenges of national cancer control plans (NCCPs) and cancer registration (Table 3). Governmental and COMISCA support for the inclusion of cancer and NCD control plans have been present in the CA-4 nations over the past decade,^26^ yet the lack of policies, budgets, training, and dedicated personnel have hindered implementation. There is evidence that the national directives and international mandates do not necessarily translate into concrete progress for the NCCPs or PBCRs, which often lack resources to carry out the activities outlined in the NCCP.^27^ Historically, cancer planning has been based within the Ministry of Health Epidemiology Division and without dedicated personnel. Public health urgencies due to natural disasters and outbreaks (eg, Chikungunya, Zika) regularly impede advancement of public sector cancer programs.

**Table 3:**
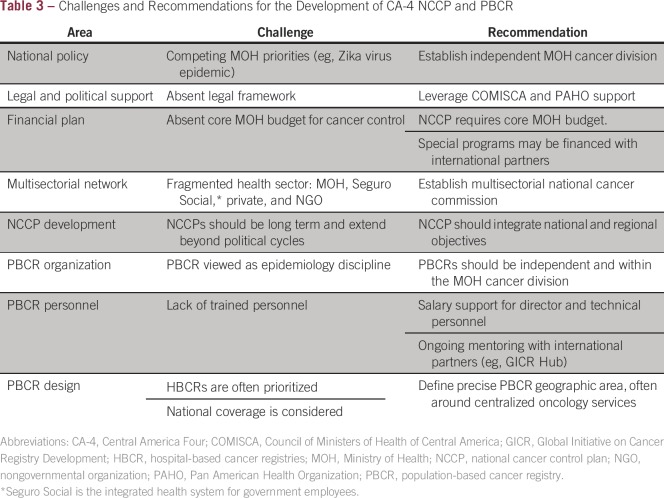
– Challenges and Recommendations for the Development of CA-4 NCCP and PBCR

The recent global recognition of the importance of cancer and NCDs in resource-limited settings is helping to broaden disease priorities. It is noteworthy that after the 2014 CA-4 Cancer Control and Bioinformatics Congress, independent cancer control entities were formed in the ministries of health of El Salvador (MINSAL) and Honduras. In May 2015, the Ministry of Health of El Salvador formed the National Department of Cancer Control and Prevention and the National Multisectorial Cancer Commission. The Honduras Ministry of Health molded the foundation (personnel, informatics) to create the Cancer Registry Division in 2015, which was formally launched in July 2016.

The legal framework for cancer registration has been lacking in the CA-4 nations, an additional challenge. Globally, certain national and MOH legal policies supporting cancer registration have proven successful in the LMIC setting.^28^ Although mandatory reporting is a desired goal in cancer registration, such mandatory reporting is not a requirement at the outset. Frequently, technical personnel leading PBCR planning have had an over-reliance on the existence of a legal framework, which may delay initial efforts.

Before the IARC-GICR initiatives to develop PBCRs, there were efforts in each CA-4 nation to develop hospital-based cancer registries (HBCRs) in the MOH public facilities and the governmental employee (Seguro Social) facilities. The development of HBCRs in the major institutions, coupled with the experience for trained personnel with specific protocols and software (eg, CANReg5, pediatric cancer), has been helpful for early capacity building and specific experience in cancer registration. Given the historical absence of specific training programs in cancer registration and the reliance on mentoring processes, these critical masses of trained personnel should be maximally leveraged.

We underscore that the development of an HBCR is not a prerequisite to advance in the establishment of a general PBCR. Although HBCRs focus on treatment and outcomes, with a significant number of variables relating to treatment protocols, the PBCRs focus on incidence and mortality, requiring fewer variables, but yet have greater precision (eg, quality coverage).^29^ In addition, the CA-4 nations have limited geographical extension, and the temptation to have a national cancer registry is understandable, following the example of Costa Rica and Uruguay. Yet, such registries would require important resources that would compete with other health priorities, face the challenge of sustainability, and be of marginal benefit of increased coverage beyond a high-quality regional PBCR.^29^ Given these considerations, it is recommended to begin with focused PBCR initiatives.

### The Design of the Initial CA-4 PBCRs

The definition of a well-defined registration area for the PBCR is a key and central aspect that needs to be established when starting to set up a PBCR.^29^ Three nations, El Salvador, Guatemala, and Honduras, have launched their initial PBCR initiatives by selecting well-defined regional areas where the implementation of a PBCR is feasible (Fig 1). In each case, given the concentration of advanced cancer care services, the regional areas are defined by the *departamentos* (states) around the respective capital cities, San Salvador, Guatemala City, and Tegucigalpa. These registries will serve as the principal PBCRs in the first phase of the renewed NCCPs. Initial seed investments are provided by international partners (eg, NCI, IARC, UICC, IAEA), either through direct agreements or via academic partners (eg, Vanderbilt University), to support planning, training, and implementation. Ultimately, support will need to transition to the respective MOHs.

With scientific sector grant funding, two rural monographic PBCRs are also being developed in El Salvador and Honduras, representing partnerships among the MOHs, local nongovernmental organizations, and US academic partners. These efforts leverage ongoing programs in these rural areas in cervical cancer in El Salvador and gastric cancer in Honduras. The rationale for these initiatives include the provision of data from rural areas, completeness data for the central PBCRs (a quality control measure), data on the flow of patients among CA-4 nations, and an additional training platform for health ministry personnel. The León, Nicaragua, HBCR is an example of the utility of an HBCR as a training platform, because it transitions to a PBCR. Lastly, with establishment of the PBCRs, and the need for survival data, improvement of vital statistics systems for mortality data are needed (eg, cause of death codification), and these additional efforts have been launched.^30,31^ Quality incidence and mortality data are also needed for the evaluation of primary and secondary prevention programs, with cervical and gastric cancers as examples in the region.^32-35^

### Early Success: CA-4 Pediatric Cancer Registration

The advancement of pediatric PBCR in each of the CA-4 countries is under way, fostered by the twinning activities and building on the experience with the POND patient data system. Although pediatric cancer represents a small percentage of the overall cancer burden, pediatric cancer registration offers a quick win for cancer registration efforts because of its feasibility, given the small numbers and centralized care, yet with a high impact on survival. The methodology of cancer registration, including the training of cancer registrars in coding practices and cancer registration software, and the production of policy and procedures, are potentially transferable to adult cancer registration and health ministry policies.

The pediatric PBCRs in Guatemala and El Salvador began in early 2014, with international partners from Dana-Farber, St Jude, and AHOPCA. In Guatemala, given the extent of the country and the existence of multiple pediatric cancer care facilities in Guatemala, the decision was to begin with a regional PBCR in Guatemala City. In El Salvador, the pediatric PBCR is a national registry, because all pediatric cancer care is delivered at a single cancer hospital in San Salvador, at Hospital Benjamin Bloom. At each site, the initiative has funded a registry medical director (part time) and a cancer registrar (full time), with international training in cancer registration for the personnel. The El Salvador and Guatemala pediatric PBCRs now register an estimated 350 and 170 patients per year, respectively.

The initial incidence data from 2014 to 2015 from the pediatric PBCRs of El Salvador and Guatemala provide proof of principal and underscore the discrepancies with GLOBOCAN estimation methods, which in the CA-4 are based on low-quality mortality data. The GLOBOCAN estimates for El Salvador in 2012 were 133 total cases, ages 0 to 14 years, or 69 cases per 100,000 population, with estimations of 60 cases of leukemia, nine cases of lymphoma, and 21 cases of CNS tumors. The observed annual incidence from the El Salvador registry is 92 cases per 100,000, including 78, 24, and 19 annual cases for these same cancers, respectively. Overall, this represents a 33% higher incidence than that predicted using GLOBOCAN (Table 4).

**Table 4:**
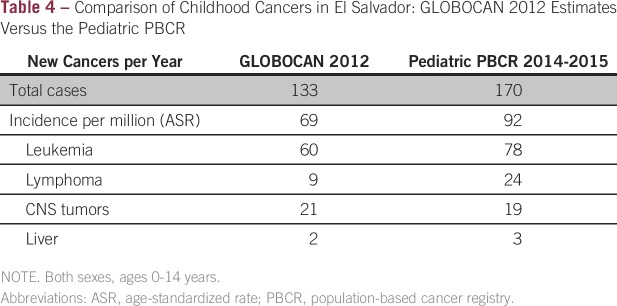
Comparison of Childhood Cancers in El Salvador: GLOBOCAN 2012 Estimates Versus the Pediatric PBCR

Incidence and survival estimates for pediatric cancers need to be improved via quality PBCRs. Two examples are germane. Three of the CA-4 countries with available mortality data by cause of death (El Salvador, Guatemala, and Nicaragua) exhibit high childhood leukemia mortality rates (under 14 years of age) that situate them among the first 30 countries in the world with the highest rates.^19^ However, the previously cited data available from AHOPCA reported 3-year event-free survival rates of standard-risk acute lymphoblastic leukemia in El Salvador to be 68.5% in 2013.^20,21^ (Note, these data conservatively included abandonment as an event rather than as a loss to follow–up.) In Guatemala, Honduras, and Nicaragua, the third most common estimated cancer in children by GLOBOCAN is liver cancer; however, in our recent PBCR data from El Salvador, liver cancers in children are rare, usually approximately 5% of the total incidence. These examples underscore the importance and value of PBCRs to provide quality data for the NCCPs and health ministry policy.

### Role and Support Through Partnerships

The specific near-term initiatives partnered with the local MOHs in cancer control and cancer registration in the CA-4 region are listed in Table 5. Cancer registration requires trained personnel and precision in the context of a fragmented health sector and informatics challenges in the LMIC setting. Data from PBCRs has contributed significantly to NCCPs and to improved understanding of disease epidemiology and etiology. Registries must follow international standards, and their personnel must adhere to established coding, classification, and quality control principles. Nations are beginning to advance their NCCPs and PBCRs, which is increasing the demand for technical training. The IARC-led GICR program aims to achieve this by recruiting and retaining local experts and by fostering mentorships to provide coordinated support for strengthening local surveillance capacity. Support through partners is essential, as has been illustrated in the case of pediatric care and registration.

**Table 5:**
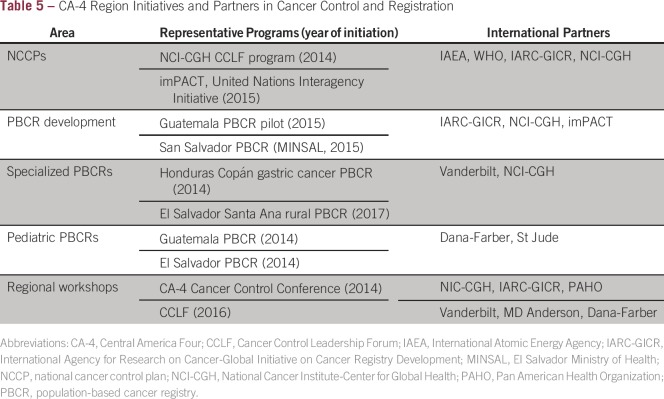
CA-4 Region Initiatives and Partners in Cancer Control and Registration

The case of Guatemala is instructive on the utility of partnerships. In the pilot phase of the Guatemala City PBCR, managed by the NCI (Instituto Nacional de Cancerologia), the HBCR is being used to network hospitals to gather information. Continuous education and mentoring is a key aspect of personnel retention. An annual regional training course is organized by the IARC-GICR Latin America Hub (Argentina), with onsite and webinar training components. Mentoring is a viable strategy to build chronic diseases research capacity.^36^ Guatemala has an established mentoring experience to increase research capacity.^37,38^ An expanded program is being planned by GICR in the context of the Latin American Hub activities, whereby longstanding PBCRs (eg, Colombia) would host LMIC faculty and staff.

In a new United Nations multiagency initiative (imPACT) involving the IAEA, WHO, and IARC, El Salvador has been preliminarily selected for in-depth assistance in the different components of cancer control. A recent workshop will provide the basis to mobilize resources, and if successful, it will provide a useful model to replicate in the CA-4 and other regions.

The NCI-CGH has partnered with US academic cancer centers (eg, Vanderbilt University, MD Anderson, Dana-Farber) via competitive funding initiatives to build cancer registration and control capacity in the CA-4 and in global LMICs. In the CA-4, these initiatives have been able to leverage existing scientific sector infrastructures, often built on specific cancers (eg, gastric, cervical), to launch PBCRs and regional cancer control initiatives, as well as serve as the basis for international networks in the region with IARC-GICR, PAHO, UICC, and other institutions.

In conclusion, the significant and growing cancer burden in the CA-4 region, dominated by the infection-associated cancers and the paucity of information, and with NCCPs under development, underscores the urgent need for cancer control and registration capacity building. Efforts are now under way to establish PBCRs and comprehensive, evidence-based NCCPs in the CA-4. There is a need for concrete and sustained governmental support to be able to implement the NCCPs’ activities, particularly cancer registration. International agencies (IARC-GICR, NCI-CGH) and academic cancer centers will continue to serve as important partners in the near term. In addition, a regional approach within the CA-4 seems both plausible and efficacious to optimally serve the region’s population of 36 million. With the international partners, the involvement of regional bodies such as COMISCA and PAHO is imperative.
